# 

**Published:** 1911-11

**Authors:** 


					﻿Vol. XXVII
NOVEMBER, 1911
No. 5
TEXAS
MEDICA
JOURNAL
'«Fuiy« looO
“Red Back”
PUBLISHED AT AUSTIN, TEXA>
F. E. DANIEL, M. D., - - - Editor and Proprietor;
PRINTED BY THE VON BOECKMANN-JONES CO.
Subscription, $1.00 a Year, in Advance. -	Single Copies, 10 Cents
Entered at the Postoffice at Austin, Texas, as second class mail matter.
				

